# AI and Machine Learning for Precision Medicine in Acute Pancreatitis: A Narrative Review

**DOI:** 10.3390/medicina61040629

**Published:** 2025-03-29

**Authors:** Sandra López Gordo, Elena Ramirez-Maldonado, Maria Teresa Fernandez-Planas, Ernest Bombuy, Robert Memba, Rosa Jorba

**Affiliations:** 1 General and Digestive Surgery Department, Maresme Health Consortium, 08304 Mataro, Spain; ebombuy@csdm.cat; 2 Unit of Human Anatomy and Embriology, Department of Morphological Sciences, Faculty of Medicine, Universitat Autònoma de Barcelona, Cerdanyola del Vallès, 08193 Barcelona, Spain; 3 General and Digestive Surgery Department, Universitary Hospital of Tarragona Joan XXIII, 43005 Tarragona, Spain; rmembai.hj23.ics@gencat.cat (R.M.); rjorba.hj23.ics@gencat.cat (R.J.); 4 Biomedicine Department, Rovira i Virgili University, 43007 Tarragona, Spain; 5 Radiology Department, Maresme Health Consortium, 08304 Mataro, Spain; mfernandezpl@csdm.cat

**Keywords:** artificial intelligence, machine learning, acute pancreatitis, severity, personalized medicine

## Abstract

Acute pancreatitis (AP) presents a significant clinical challenge due to its wide range of severity, from mild cases to life-threatening complications such as severe acute pancreatitis (SAP), necrosis, and multi-organ failure. Traditional scoring systems, such as Ranson and BISAP, offer foundational tools for risk stratification but often lack early precision. This review aims to explore the transformative role of artificial intelligence (AI) and machine learning (ML) in AP management, focusing on their applications in diagnosis, severity prediction, complication management, and treatment optimization. A comprehensive analysis of recent studies was conducted, highlighting ML models such as XGBoost, neural networks, and multimodal approaches. These models integrate clinical, laboratory, and imaging data, including radiomics features, and are useful in diagnostic and prognostic accuracy in AP. Special attention was given to models addressing SAP, complications like acute kidney injury and acute respiratory distress syndrome, mortality, and recurrence. AI-based models achieved higher AUC values than traditional models in predicting acute pancreatitis outcomes. XGBoost reached an AUC of 0.93 for early SAP prediction, higher than BISAP (AUC 0.74) and APACHE II (AUC 0.81). PrismSAP, integrating multimodal data, achieved the highest AUC of 0.916. AI models also demonstrated superior accuracy in mortality prediction (AUC 0.975) and ARDS detection (AUC 0.891) AI and ML represent a transformative advance in AP management, facilitating personalized treatment, early risk stratification, and allowing resource utilization to be optimized. By addressing challenges such as model generalizability, ethical considerations, and clinical adoption, AI has the potential to significantly improve patient outcomes and redefine AP care standards globally.

## 1. Introduction

Acute pancreatitis (AP) is a common gastrointestinal disorder characterized by acute inflammation of the pancreas. It remains one of the leading causes of hospital admissions worldwide, with an incidence rate ranging from 13 to 45 cases per 100,000 people annually [1]. In the United States alone, AP accounts for over 270,000 hospitalizations per year, resulting in significant healthcare costs exceeding USD 2.5 billion [1]. AP can range in severity from mild, self-limiting disease to severe acute pancreatitis (SAP), which is associated with persistent organ failure, pancreatic necrosis (5–10%), and high mortality rates, reaching 36–50% in severe cases [2].

The 2012 Revised Atlanta Classification categorizes AP into three types based on severity: mild (no organ failure or complications), moderately severe (transient organ failure or local complications), and severe (persistent organ failure) [2]. While mild cases of AP often resolve within a week, severe forms can lead to multi-organ failure, sepsis, and prolonged hospitalization. Early and accurate risk stratification is crucial for managing AP, as it guides interventions and resource allocation, ultimately improving patient outcomes.

**Figure 1:**
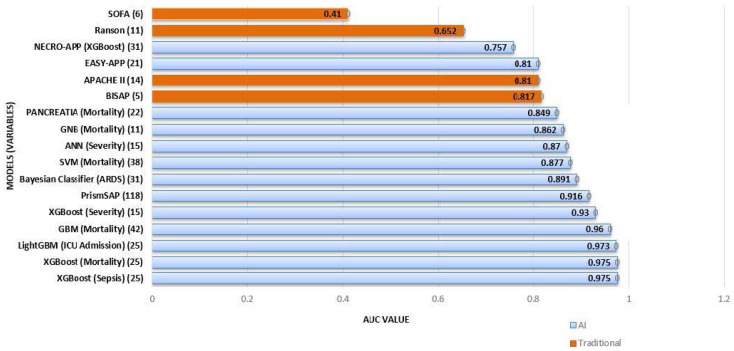
Comparison of traditional models vs. AI-based models for predicting pancreatitis severity and complications. Models with the higher AUCs.

Traditional scoring systems, such as the Ranson criteria, the Bedside Index for Severity in Acute Pancreatitis (BISAP), and the Acute Physiology and Chronic Health Evaluation II (APACHE II), have long been used to predict severity and prognosis. However, these methods rely on predefined clinical and laboratory parameters, often exhibiting limited accuracy in the early stages of the disease. For instance, Ranson and BISAP scores have sensitivities of 0.95 and 0.67, respectively, for severity prediction, with area under the curve (AUC) values of 0.95 and 0.94. Mortality sensitivity was reported at 0.89 for Ranson and 0.77 for BISAP, with AUC values of 0.91 and 0.92, respectively [3]. In Figure 1, highest AUCs are showed for the different prediction targets. Additionally, these models are static, relying on baseline or limited follow-up data, which restricts their performance in complex clinical settings. Imaging-based assessments, such as the Balthazar CT grading system and the Modified Computed Tomography Severity Index (MCTSI) [4], also play a role in severity evaluation but are subject to interobserver variability.

Figure 1 shows the highest AUC of the different models according to the prediction of each one. A differentiation has been made between classic models and those that use AI (different colors). The number of variables in each model are shown in parentheses.

In recent years, artificial intelligence (AI) and machine learning (ML) have gained prominence in medical research and clinical practice. AI-driven algorithms can analyze vast and complex datasets, including clinical, laboratory, and imaging data, identifying intricate nonlinear relationships that traditional models may overlook. ML, a subset of AI, focuses on developing algorithms that learn from data to improve prediction accuracy over time. AI has the potential to enhance prognostic capabilities by detecting subtle patterns within biochemical parameters and disease outcomes, making it a valuable tool for improving AP risk stratification and management.

Compared to conventional scoring systems (Table 1), AI-driven models dynamically process large-scale clinical data, integrating multiple data modalities such as radiomics, electronic health records, and biochemical markers. These models facilitate early diagnosis, risk stratification, and real-time prediction of complications, such as acute kidney injury (AKI), acute respiratory distress syndrome (ARDS), and mortality [5]. Some AI-based applications, such as EASY-APP and NECRO-APP, have been specifically developed to assess severity and pancreatic necrosis, respectively. Meanwhile, PrismSAP integrates radiomics and electronic health data for superior prognostic accuracy.

AI-based approaches vary in complexity, interpretability, and data requirements, yet their adaptability makes them suitable for diverse research settings, real-time predictions, and personalized insights. The integration of AI into AP management has the potential to improve clinical decision making, optimize patient outcomes, and refine diagnostic and therapeutic strategies [6].

The objective of this review is to synthesize current knowledge on AI-based tools in AP and evaluate their predictive capabilities compared to traditional methods. Understanding the advantages of AI-driven models will contribute to enhanced clinical decision making, ultimately improving patient care and treatment outcomes.

## 2. Diagnostic and Differential Diagnostics in Acute Pancreatitis

AP diagnostic is based on a combination of clinical presentation, amylase elevation in a blood test, and imaging studies. The diagnostic is confirmed when at least two of the previous three criteria are met [1].

In case of diagnostic uncertainty, imaging such as computed tomography (CT) can help rule out other causes of pain or confirm AP. However, an initial CT is not recommended for diagnosing AP; it should be reserved for diagnostic uncertainty, clinical deterioration, or the need to assess disease severity [1]. Imaging also plays a crucial role to identify the severity and potential complications of AP, such as necrosis or fluid collections [4]. While AP diagnosis is primarily based on clinical and laboratory findings, imaging has become indispensable for accurate assessment and management. In this context, AI and advanced imaging techniques are gaining increasing relevance. AI can enhance image analysis and interpretation, improving diagnostic accuracy and supporting clinical decision making.

The pancreas varies significantly between individuals in terms of shape, size, and positioning; there are also some anatomical variabilities and its position, deep in the abdomen, makes it difficult to image clearly, with extra difficulties when the tissue is inflamed like in AP [7]. To overcome these limitations, radiologists increasingly employ segmentation techniques, which help in the analysis of pancreatic structures, thereby improving diagnostics and accuracy [8].

AI models that use CT scan data can not only detect the presence of AP but also assess the severity of AP. In Chi Zhang’s study [4], two key modules were created. 1 Acute Pancreatitis Classifier, designed to detect pancreatitis based on CT images, and 2 Lesion Segmentation Module, used to identify affected areas in the pancreas (e.g., swelling and abscess) [4]. The results about the diagnostic model exhibit an excellent efficacy in accurately recognizing AP and assess its severity. Including specific performance metrics, such as AUC values or accuracy rates, would provide concrete evidence of the models’ effectiveness and further strengthen the argument for their clinical applicability.

Radiomics, the field in medical imaging that extracts quantitative features from medical images, is not only used for the diagnosis of AP but also to predict complications or the progression of AP [9]. Radiomics can also be integrated with clinical and laboratory data to create multimodal diagnostic models that outperform single-modality approaches. This aspect will be addressed more thoroughly in a later section of this review.

Differentiating AP from conditions such as functional abdominal pain or chronic pancreatitis can be challenging. Radiomic analysis of CT images has proven valuable in distinguishing between these entities. Notably, IsoSVM, a model combining Isomap and support vector machine algorithms, achieved an accuracy of 82.1% in diagnosing these conditions [10]. If the capacity of AI is integrated into the flows of daily clinical practice, real-time information will be obtained, improving precision and patient management.

## 3. Prediction of Severity and Complications

In mild acute pancreatitis, complications are rare and recovery typically occurs within less than a week. In contrast, SAP (20–30% of cases) is associated with complications such as pancreatic necrosis or organ failure, which can lead to a high mortality rate of 13–35% [2,11,12]. Early identification of SAP is crucial for improving outcomes, as timely intervention can mitigate complications such as organ failure, necrosis, and systemic inflammation [12,13].

Traditional scoring systems, including APACHE II, Ranson, and BISAP, offer predictive capabilities [3] but frequently lack the accuracy and timeliness required, especially during the critical early phase of the disease. ML models provide a more advanced approach, integrating large datasets, including vital signs, laboratory parameters, and imaging data, to enhance predictive accuracy in real time [14]. AI-driven models offer more personalized risk assessments and improved clinical decision making.

The EASY-APP model, which integrates clinical parameters available at admission, including respiratory rate, body temperature, abdominal muscular reflex, gender, age, and glucose level, demonstrated an ROC-AUC of 0.81 for predicting SAP at the first hours of admission [15]. Similarly, the Artificial Neural Network (ANN) model, focusing exclusively on initial triage using baseline clinical variables without requiring laboratory or imaging data, achieved an AUC of 0.849 for mortality and 0.783 for persistent organ failure, making it particularly valuable in resource-limited settings [16].

PANCREATIA demonstrates comparable performance, with AUCs up to 0.849 for mortality, 0.786 for ICU, and 0.783 for persistent organ failure using only early-stage data. This makes PANCREATIA particularly valuable in resource-limited settings or emergency situations where rapid and accurate triage is crucial for guiding referrals, initiating timely interventions, and optimizing patient management. Its streamlined approach offers a practical solution for early risk stratification in AP [16]. Another study demonstrating a higher AUC for organ failure prediction is the one conducted by Lin and colleagues, achieving an impressive AUC of 0.915 with Random Forest (RF) [17]. This result contrasts with the findings of Qiu and colleagues, who, in their 2019 study involving 263 patients, highlighted the potential of ML for predicting organ failure but reported lower performance (AUC 0.832–0.840) [18].

Extreme Gradient Boosting (XGBoost) has also shown high predictive performance, with AUC of 0.92–0.93 within the first 48 h, outperforming ANN (AUC: 0.87) and conventional models like BISAP (AUC: 0.74) and APACHE II (AUC: 0.80) [19]. Key predictive variables identified include blood urea nitrogen (BUN), pleural effusion, and systemic and high-density lipoprotein cholesterol (HDL-C), which were the three most influential variables. Other important predictors included systemic inflammatory response syndrome (SIRS), C-reactive protein (CRP), and markers of systemic inflammation and organ dysfunction [12,19].

AI-based models have demonstrated even greater potential when integrated into the hospital information system (HIS). Chang et al. developed models using Light Gradient Boosting Machine (LightGBM) and XGBoost, achieving AUCs of 0.961 for sepsis, 0.973 for ICU admission, and 0.975 for mortality, surpassing traditional scoring systems [20]. Other ML models like GBDT had better prediction of sepsis than the classical models, with an AUC of 0.985 [21].

Additionally, multimodal approaches, like the PrismSAP model, which combine clinical data, electronic health records, and radiomics, have significantly improved predictive accuracy, achieving an AUC of 0.916 in external validation [14].

Real-time AI predictions hold significant clinical value, enabling rapid risk stratification in emergency settings. Models leveraging Least Absolute Shrinkage and Selection Operator (LASSO) regression have identified key predictive features such as age, diabetes, neutrophil counts, hematocrit, and systemic inflammatory response syndrome (SIRS) [14]. AI also aids in resource allocation, facilitating early identification of high-risk patients and optimizing ICU admissions [13].

Despite these advancements, challenges remain in the widespread adoption of AI in SAP management. Model generalizability across different populations and healthcare settings requires further validation through large-scale, multicenter studies [12]. Additionally, integrating AI tools into routine clinical workflows requires user-friendly interfaces and robust interpretability features to ensure clinician adoption and trust. For instance, in the development of multimodal models such as PrismSAP, techniques such as Local Interpretable Model-Agnostic Explanations (LIME) and Gradient-weighted Class Activation Mapping (Grad-CAM) were employed to improve transparency and provide actionable insights into the variables driving predictions [12,14].

In conclusion, AI and ML have revolutionized SAP prediction and management by offering higher predictive accuracy, integrating diverse data sources, and achieving real-time decision making. However, ongoing research and validation are essential to ensure their full potential is realized and effectively implemented in clinical practice.

## 4. Radiomics in Acute Pancreatitis

CT is the most commonly used imaging modality for assessing AP, identifying necrosis, fluid collections, and indirect signs of infection. However, MRI is emerging as a valuable alternative, offering advanced techniques such as diffusion-weighted imaging, that can differentiate between viable and necrotic pancreatic tissue with high sensitivity and specificity by measuring apparent diffusion coefficient values, which correlate with inflammation and tissue injury. T1 and T2 mapping add further diagnostic capabilities by quantifying changes in tissue relaxation times, reflecting pancreatic edema and fibrosis. Radiomics can also use MRI data and use these to predict AP severity and complications, achieving then a higher AUC than the traditional clinical models. These advanced imaging techniques, combined with ANN for data integration, can identify risk stratification, offering a significant advantage over conventional methods. The findings underscore the potential of MRI to revolutionize the assessment and management of AP, moving beyond the limitations of CT imaging [9].

AI is also enhancing CT assessment by identifying indicators of AP severity [22]. A study by Hongyin Liang analyzed 1798 CT scans using two classification systems: the Computed Tomography Severity Index (CTSI) and the 2012 revised Atlanta classification. The AI model achieved an AUC-ROC of 0.980 when trained with CTSI-labeled scans, demonstrating the effectiveness of CNNs in extracting severity-related imaging data [23].

Integrating imaging with clinical and analytical data further improves predictive accuracy. The PrismSAP model, which combines electronic health records, radiomics (3D CT scans), and deep learning, outperformed traditional scoring systems like Ranson and BISAP, achieving an external test AUC of 0.916 [14]. Similarly, the NECRO-APP model, designed for early necrosis prediction, achieved an ROC-AUC of 0.757 using XGBoost Classifier, highlighting its sensitivity in identifying patients at risk of pancreatic necrosis, a key factor in SAP severity [24].

## 5. Acute Respiratory Distress Syndrome

Acute respiratory distress syndrome (ARDS) is a complication that occurs in SAP and it is responsible for about 60% of death within the first week of disease onset [25]. For this reason, early identification is important; some authors, like Kang Zou et al. [25]., found the importance of ANN and logistic regression in prediction of ARDS. Key predictive factors included were BISAP score, procalcitonin, prothrombin time, and serum calcium levels Four ML models like support vector machine (SVM), Ensembles of Decision Trees (EDTs), Bayesian Classifier (BC), and a nomogram model that used different variables than the first manuscript like PaO2, C-reactive protein, procalcitonin, lactic acid, etc., were combined. The BC achieved the highest area under the curve (AUC) of 0.891 compared to the ANN model, which had an AUC of 0.853 [26].

All of these models are examples of uses to detect ARDS in SAP and prevent mortality.

## 6. Acute Kidney Injury

Acute kidney injury (AKI) is a severe complication of AP, as a significant impact in the prognosis, and is associated with a high mortality rate. ML has also been utilized to identify high-risk patients who are likely to develop AKI [27,28].

The systemic inflammatory markers and clinical variables included in these ML models are critical, as they should specifically encompass those known to be associated with AKI in the context of AP [29,30].

GBM models achieved some of the highest performance metrics, with AUC values ranging from 0.814 to 0.867 across training and test datasets, as reported using the Medical Information Mart for Intensive Care IV (MIMIC-IV database), which is a publicly accessible, deidentified database containing detailed clinical data from over 500,000 ICU admissions from 2008 to 2019 [27]. Similarly, other ML methods, such as RF with an AUC of 0.812 and support vector machine (AUC 0.810), demonstrated a high predictive capacity in the same study [27]. Comparatively, neural networks showed lower AUC values of 0.688, reflecting potential limitations in certain applications [27]. In another study, logistic regression models for AKI prediction achieved AUC values between 0.763 and 0.828, depending on the dataset and feature inclusion [30]. The capacity of AI and ML in kidney damage can improve clinical decisions and improve outcome by early detection or identifying patients susceptible to AKI.

## 7. Survival and Mortality

Traditional scoring systems, such as Ranson and SOFA, often require extensive data collection and exhibit limited predictive accuracy, with AUCs of 0.652 and 0.401, respectively, for hospital mortality prediction [31]. In contrast, ML and AI have demonstrated superior predictive capabilities [32]. For example, ANN developed using the MIMIC-III database (ICU admissions from 2001 to 2012) achieved an AUC of 0.769 for in-hospital mortality prediction, outperforming logistic regression and traditional scores while requiring fewer input variables [31,33]. ANN was able to predict death, with an accuracy of 97.5%, and was more accurate than APACHE II scoring system or GS score at 48 h [19]. Another study using GNB reported AUCs of 0.840 and 0.862 in internal and external validations, respectively, identifying red cell distribution width and blood oxygen saturation as critical predictors [34]. Anjuli and collaborators describe a higher AUC of 0.96 with the GBM model [35].

Generative models can also be used to analyze mortality. They are designed to create synthetic data that mimic the characteristics of the original dataset, addressing challenges like data imbalance and limited sample sizes. In contrast, classic predictive AI models analyze existing data to make predictions, classify outcomes, or identify patterns directly relevant to the problem at hand, such as predicting mortality or disease severity [33].

These generative models included Conditional Tabular Generative Adversarial Networks (CTGAN), which excel at synthesizing high-quality tabular data while preserving feature distributions, and CopulaGAN, designed to maintain statistical dependencies within the data. Additionally, other augmentation approaches, such as Variational Autoencoders (VAEs) and Synthetic Minority Over-Sampling Technique (SMOTE), were utilized to enrich the dataset and improve model performance [33]. Chang and colleagues found that XGBoost showed the highest AUC for mortality (AUC 0.975) [20]. These findings underscore the transformative potential of ML and AI in optimizing mortality prediction in AP; they offer an accurate identification of high-risk patients to guide clinical management in AP.

## 8. Recurrence

Recurrent AP is defined as two or more distinct episodes of AP separated by a period of complete clinical and biochemical resolution, typically lasting at least three months, with each episode meeting the diagnostic criteria for AP. Approximately 21% of patients experience recurrent AP after the initial episode, with recurrence rates varying by etiology: 12% for biliary pancreatitis, 30% for alcoholic pancreatitis, 25% for idiopathic pancreatitis, and 30% for hypertriglyceridemia-induced pancreatitis, highlighting the importance of addressing underlying causes to reduce recurrence risk [36].

ML offers promising possibilities for managing the recurrence of AP by improving diagnostic accuracy and predicting outcomes. Studies use a combination of radiomic features extracted from CT images—such as texture, shape, and intensity data—and clinical information, including patient demographics, history of recurrences, and pancreatic changes [10,37]. These models include advanced algorithms like IsoSVM to analyze subtle patterns in imaging and clinical variables. IsoSVM achieved in the recurrent AP group a sensitivity of 95%, specificity of 78%, and an AUC of 0.88 [10]. We have seen before how XGBoost has proven to be a powerful tool in predicting outcomes and complications in AP but these are also useful for better identification of patients at risk of recurrence, addressing challenges like normal imaging post-episode and allow tailored follow-up and early interventions to prevent progression to chronic pancreatitis [38].

## 9. Surgical Timing

Surgery or an interventional approach in AP usually is indicated in patients with infected necrosis and several studies have cheeked the perfect time to perform the surgery or the intervention to reduce complications and achieve a better recovery. Recent advancements in ML have demonstrated potential in guiding these decisions. For example, Lan et al. [39] included logistic regression (LR), support vector machine (SVM), and RF for predicting the need for early versus delayed surgery (before or after 4 weeks). They incorporate clinical parameters such as interleukin-6 (IL-6) levels, CRP, infected necrosis, and duration of organ failure. Accuracy of LR, SVM, and RF was 0.71, 0.78, and 0.80, respectively. Further refinement by Luo et al. introduced an RNN model that emphasized the dynamic evolution of clinical features over time, yielding an AUC of 0.70 for predicting intervention timing [40]. These models offer promising tools for personalizing surgical decisions in AP, leveraging ML to improve the precision in surgical timing.

## 10. Discussion

The integration of AI and ML into the management of AP represents both the present and the future, offering enhanced diagnostic accuracy, early risk stratification, and optimized treatment strategies. Through this review, the potential applications of AI in AP, including severity prediction and complication management, have been explored, while comparing its performance to traditional scoring systems and clinical methods.

Table 2 outlines the different models and the highest AUC for the different prediction targets.

In most studies focusing on the application of ML and AI in AP, traditional scoring systems such as Ranson and APACHE II consistently exhibit AUC values below 0.9 for predicting outcomes such as severity and mortality. Traditional models are often less effective in early phases of the disease. Only Zhu and colleagues [3] reported an AUC of 0.95 for the Ranson score in predicting the severity of AP. This exceptional result is an outlier compared to the findings of most ML-focused studies, which highlight the superior predictive capacity of AI models [5,19]. These ML models frequently achieve AUC values exceeding 0.9 in AP, demonstrating their ability to outperform traditional scoring systems by integrating complex datasets and offering real-time predictive insights. Zhu’s findings underscore the need for further investigation into the performance of traditional methods in diverse populations and clinical settings, while emphasizing AI’s potential to redefine risk prediction in AP.

A key distinction between AI models and classical scoring systems is that AI models integrate additional factors such as analytical variables, clinical parameters not included in traditional models, and image assessment, all of which are crucial in the evolution of AP. AI can facilitate the early identification of high-risk patients, detect AKI or ARDS, and assist in optimizing the timing for interventions, potentially leading to significant reductions in morbidity and mortality.

Similarly, multimodal approaches, exemplified by the PrismSAP model, highlight the value of integrating diverse data types—clinical, imaging, and radiomics—to achieve an AUC of 0.916, outperforming single-modality models and traditional scoring systems. These findings emphasize the potential of AI including radiomics in the management of AP evolution.

In complications such as AKI and ARDS, ML has a high utility. GBM have achieved AUCs up to 0.867 in predicting AKI and BC AUC up to 0.891 for ARDS prediction. Given the pivotal role of AP-related complications in disease progression and the need for intensive care admission, early diagnosis and improved management of these complications can optimize intensive care unit (ICU) admissions, enhance mortality prediction, and ultimately reduce mortality rates [34].

Another significant application of AI is its role in determining optimal surgical timing for infected necrosis in AP. AI models that incorporate dynamic clinical parameters, such as IL-6 levels and the duration of organ failure, have shown impressive performance, achieving AUC values as high as 0.8. This achieves the personalization of surgical decision making, a topic also explored by other researchers who studied the outcomes of early versus delayed surgery, particularly around the 4-week threshold. The combination of precise necrosis diagnosis and optimal surgical timing could enhance the management of AP complications, promoting faster recovery with lower morbidity [39].

Moreover, radiomics-driven AI tools have advanced the use of imaging modalities such as CT and MRI; the integration of these two modalities enhances the accurate identification of pancreatic changes during the AP process and improves severity prediction. For instance, the combination of CT-based radiomics with DL models has achieved AUC values as high as 0.980 for AP severity prediction, while MRI radiomics continues to emerge as a powerful complementary modality [23].

Despite these advancements, several challenges remain. One significant limitation is the generalizability of AI models. Many studies are restricted to single-center data or lack external validation, raising concerns about their applicability in diverse healthcare settings. Furthermore, AI models often require large, high-quality datasets for training, which may not always be available in resource-constrained environments and healthcare centers. Smaller medical centers, in particular, struggle with data collection due to the difficulty of recording them. A potential solution to this issue lies in fostering multicenter collaborations, implementing standardized data collection protocols, and the development of robust models capable of reliable performance across populations.

Another challenge lies in the integration of AI into clinical workflows. While models such as EASY-APP and PANCREATIA are user-friendly at admission, many AI tools lack interpretability, making clinicians hesitant to rely on them. AI applications should be designed for ease, ensuring that their algorithms’ outputs and recommendations are transparent and comprehensive for clinical adoption. Additionally, ethical considerations, including data privacy and algorithmic bias, must be addressed to ensure equitable and secure implementation of AI in healthcare.

AI and ML also hold promise for predicting AP recurrence and advancing personalized treatment strategies. For instance, XGBoost models analyzing CT-derived radiomic features have demonstrated potential in identifying patients at risk of recurrence [10]. This capability facilitates personalized follow-up and preventive interventions, helping to mitigate the progression to chronic pancreatitis if the underlying cause is not addressed. Future research should prioritize expanding these applications to address unmet needs in AP management, such as recurrence prediction and the development of targeted therapies.

While AI and ML offer substantial improvements in the diagnosis, risk stratification, and management of AP, their adoption must be balanced with clinical expertise. AI should complement rather than replace medical judgment, serving as a valuable tool that enhances, rather than dictates, decision making. The successful integration of AI into AP management will require a collaborative approach, combining technological advancements with the irreplaceable insights of experienced clinicians to ensure optimal patient outcomes.

## 11. Conclusions

The integration of AI and ML into the management of AP represents a transformative step forward in clinical practice. These technologies demonstrate superior accuracy in predicting severity, complications, and mortality, surpassing traditional scoring systems. By a personalization of treatment strategies, identifying the best treatment times and detecting complications, AI has a very strong influence regarding the ability to improve the evolution and prognosis in AP. However, challenges such as model generalizability, ethical considerations, and clinical adoption must be addressed to fully realize their potential. With continued innovation, collaboration, and validation, AI and ML promise to redefine the standard of care for AP and transform clinical decision making.

## 12. Future Directions

AI and ML has some future directions in AP as follows.

- Model Generalizability

Current AI models often rely on single-center datasets, limiting their applicability across diverse populations and healthcare systems. Future efforts should prioritize the development of large, multicenter, and geographically diverse datasets to improve the robustness and generalizability of these models. Such initiatives would ensure that AI tools are adaptable to various clinical environments and patient demographics.

- Multimodal Data Integration

The fusion of data from multiple sources, including clinical, laboratory, imaging, genomic, and even wearable-device data, holds great promise for improving prediction accuracy and enabling personalized medicine. Future research should explore the integration of advanced omics data (e.g., proteomics and metabolomics) with radiomics and electronic health records to create comprehensive models that capture the multifactorial nature of AP.

- Real-Time Clinical Applications

While AI models have demonstrated strong predictive capabilities, their seamless integration into clinical workflows remains a challenge. Future directions should focus on developing real-time, user-friendly AI tools that can be embedded into hospital information systems to provide instantaneous risk assessments, enabling timely and evidence-based decision making. Professionals must become accustomed to and familiar with the use of big data in order to leverage the benefits of AI.

- Personalized Medicine and Predictive Analytics

AI has the potential to revolutionize personalized treatment based on patient-specific data. Predictive analytics could identify optimal treatment plans, predict responses to therapies, and prevent complications such as recurrent AP or progression to chronic pancreatitis, being more personalized.

- Advancing Imaging Techniques

Radiomics and advanced imaging modalities, such as MRI with diffusion-weighted imaging (DWI) and T1/T2 mapping, are underutilized but hold significant potential for predicting AP severity and complications. Future work should focus on incorporating these imaging techniques into AI models to enhance diagnostic precision and prognostics in some specific situations. The fusion of CT and MRI images could help if it is necessary.

- Ethical and Regulatory Considerations

As AI adoption expands, addressing ethical issues such as data privacy, algorithmic bias, and equitable access will be paramount. Future research should also involve establishing clear regulatory frameworks and validation protocols to ensure safe and transparent implementation of AI in clinical practice.

- Expanding Applications Beyond Prediction and global health

AI applications in AP are currently focused on severity prediction and complication management. Future research should explore novel uses, such as predicting long-term outcomes, optimizing timing for surgical interventions, and integrating AI into post-discharge monitoring to improve follow-up care and recurrence prevention.

Simplified, cost-effective AI models could play a crucial role in bridging healthcare disparities and needs. Continuous innovation, validation, and collaboration will ensure these technologies not only enhance patient outcomes but also redefine the standard of care in AP management.

## Figures and Tables

**Table 1:** Characteristics of traditional models and AI models.

Characteristic	Traditional Models (Ranson, BISAP, APACHE II, and SOFA)	AI-Based Models
Number of Variables	5–12	15–116
Type of Data Used	Clinical + Lab	Clinical + Lab + Imaging + Radiomics
Predictive Accuracy (AUC)	0.65–0.82	0.85–0.97
Adaptability	Fixed criteria, no adaptation to real-time data	Dynamic, learns from new data
Real-Time Analysis	No	Yes
Use of Imaging Data	Limited (CTSI only)	Extensive (Uses advanced radiomics and multimodal data)
Complexity	Low (Simple scoring systems)	High (Uses complex algorithms)
Interpretability	High (Easily interpretable)	Moderate (Requires explainability techniques)
Clinical Integration	Widely used, easy to apply	Emerging, requires integration with hospital systems
Limitations	Lower accuracy, limited data integration	Requires large datasets, potential bias, regulatory hurdles

**Table 2:** Models in acute pancreatitis. Models with the highest AUCs.

Model	Prediction Target	Patients Included (N)	Total Variables (N)	Statistically Significant Features for the Prediction Target and Model	AUC Value	Reference
EASY-APP	Severity	1184	21	Age, gender, RR, BT, AMR, glucosa	0.81	Kui et al. [15]
NECRO-APP (XGBoost)	Necrosis	2387	31	PCR, Glucose, Total WBC, Hb, RBC, LDH	0.757	Kiss et al. [24]
PrismSAP	Severity	1221	9 + 107 (radiomics)	PE, SIRS, HT, RDW	0.916	Yin et al. [14]
XGBoost	Severity	648	15	BUN, PE, HDL-C	0.93	Lu et al. [12]
ANN	Severity	648	15	Glucose, albumin, PE	0.87	Lu et al. [12]
XGBoost	Sepsis	8274	25	ND	0.975	Chang [20]
XGBoost	Mortality	8274	25	ND	0.975	Chang et al. [20]
LightGBM	ICU Admission	8274	25	Amylase	0.973	Chang et al. [20]
PANCREATIA	Mortality	594	22	Advanced age, ASA, tachycardia, satO_2_/FiO_2_, BUN	0.849	Villasante et al. [16]
Support vector machine (SVM)	Mortality	534	38	WBC, platelet count, temperature, age, BUN, RDW, SpO_2_, Hb	0.877	Cai et al. [32]
Bayesian Classifier (BC)	Acute Respiratory Distress	460	31	PaO_2_, PCR, Procalcitonin, Calcium, NRL, WBC, LA, Amylase	0.891	Zhang et al. [26]
Gaussian Naive Bayes (GNB)	Mortality	1281	11	MCDW, satO_2_/FiO_2_, SIRS, BUN	0.862	Ren et al. [34]
Gradient Boosting Machine (GBM)	Mortality	97027	42	Increasing age	0.96	Anjuli K Luthra et al. [35]
Random Forest (RF)	Organ Failure	143	7 + 4 (radiomics)	HDL-C, Calcium, amylase, Apo-AI, lipasa	0.915	Lin et al. [41]
Auto-encoder (EA)	Septic shock	604	11	Heart Rate, respiratory rate, lactate, base excess, cystatin	0.900	Xia et al. [42]
BISAP	Severity	8274	25	ND	0.817	Chang et al. [20]
Ranson	Severity	337	12	ND	0.652	Ding et al. [31]
APACHE II	Severity	664	10	SIRS, hypotension, Age > 60	0.81	Mofidi et al. [19]
SOFA	Severity	337	12	ND	0.41	Ding et al. [31]

RR: respiratory rate; BT: body temperature; AMR: abdominal muscular reflex; PCR: C-reactive protein, WBC: white blood cells; Hb: hemoglobin; RBC: red blood cells, LDH: lactate dehydrogenase; PE: pleural effusion, SIRS: systemic inflammatory response syndrome; HC: hematocrit, RDW: red cell distribution width; BUN: blood urea nitrogen; PE: pleura effusion; HDL-C: high-density lipoprotein cholesterol; ASA: high American Society of Anesthesiologists score; satO_2_/FiO_2_: lower oxygen saturation/inspired oxygen fraction; MCDW: maximum red cell distribution width; PaO_2_: partial pressure of oxygen; NLR: neutrophil/lymphocyte ratio; LA: lactic; Apo-AI: apolipoprotein AI; ALT: alanine aminotransferase; ERCP: endoscopic retrograde cholangiopancreatography; ND: no data available.

## Acronyms and Definitions

AI	Artificial Intelligence
AKI	Acute Kidney Injury
ANN	Artificial Neural Network
APACHE	Acute Physiology and Chronic Health Evaluation Score
ARDS	Acute Respiratory Distress Syndrome
AUC	Area Under the Curve
BC	Bayesian Classifier
BISAP	Bedside Index for Severity in Acute Pancreatitis
BUN	Blood Urea Nitrogen
CNN	Convolutional Neural Network
CRP	C-Reactive Protein
CT	Computed Tomography
CTGAN	Conditional Tabular Generative Adversarial Networks
CTSI	Computed Tomography Severity Index
DL	Deep Learning
EA	Auto-encoder
EASY-APP	Early AI Model for Pancreatitis Severity Prediction
GBM	Gradient Boosting Machine
GNB	Gaussian Naive Bayes
ICU	Intensive Care Unit
LASSO	Least Absolute Shrinkage and Selection Operator
LightGBM	Light Gradient Boosting Machine
MCTSI	Modified Computed Tomography Severity Index
ML	Machine Learning
MRI	Magnetic Resonance Imaging
NECRO-APP	AI-Based Necrosis Prediction Model
PANCREATIA	Predictive Model for Mortality in Acute Pancreatitis
PrismSAP	Multimodal AI Model for Acute Pancreatitis
RF	Random Forest
ROC	Receiver Operating Characteristic
SANRA	Scale for the Assessment of Narrative Review Articles
SIRS	Systemic Inflammatory Response Syndrome
SMOTE	Synthetic Minority Over-Sampling
SOFA	Sequential Organ Failure Assessment Score
SVM	Support Vector Machine
VAEs	Variational Autoencoders
XGBoost	Extreme Gradient Boosting
